# Three Layers of Personalized Medicine in the Use of Sirolimus and Its Derivatives for the Treatment of Cancer

**DOI:** 10.3390/jpm13050745

**Published:** 2023-04-27

**Authors:** Andres Delgado, Steven Enkemann

**Affiliations:** 1 Aultman Hospital/NEOMED Program 1, Canton, OH 44710, USA; andresd0370@gmail.com; 2 Edward Via College of Osteopathic Medicine, 350 Howard St., Spartanburg, SC 29303, USA

**Keywords:** rapamycin, cytochrome, p450 enzyme, chemotherapy, Sirolimus, Everolimus, Ridaforolimus, Temsirolimus

## Abstract

Rapamycin and its derivatives are mTOR inhibitors which are FDA-approved for use as immunosuppressants and chemotherapeutic agents. These agents are currently approved to treat renal cell carcinomas, soft tissue sarcomas, and other rare tumors. As tumor treatment paradigms are moving away from organ-based drug selection and moving towards tumor characteristics for individualized treatment it is important to identify as many properties as possible that impact the efficacy of the rapalogues. A review of the current literature was conducted to identify enzymes involved in the metabolism of Sirolimus, Everolimus, Ridaforolimus, and Temsirolimus along with characteristics of tumors that predict the efficacy of these agents. This review also sought to establish whether the genetic characteristics of the patient might influence the activity of the rapalogues or lead to side effects from these agents. Current evidence suggests that tumors with mutations in the mTOR signal transduction pathway are sensitive to rapalogue treatment; the rapalogues are metabolized by cytochromes such as CYP3A4, CYP3A5, and CYP2C8 and transported by ABC transporters that are known to vary in activity in individuals; and that tumors can express these transporters and detoxifying enzymes. This results in three levels of genetic analysis that could impact the effectiveness of the mTOR inhibitors.

## 1. Introduction

The newest paradigm for cancer treatment is to stimulate the immune system. Ironically, another class of agents in the arsenal against cancer inhibits the immune system. Rapamycin, and its analogs, are widely used to suppress the immune system in patients who have had organ transplants. They have also been used to treat certain cancers. The original compound was isolated from the soil bacterium, Streptomyces hygroscopicus, which was first isolated from Easter Island, known natively as Rapa Nui [1]. As the isolated compound was initially shown to have antimicrobial activity the compound was named rapamycin. The rapamycin family now includes Sirolimus (the common chemical name for rapamycin), Everolimus, Temsirolimus, and Ridaforolimus, formerly known as Deforolimus. Sirolimus is primarily used as an immunosuppressant to prevent transplanted organ rejection, but it has also been approved to treat lymphangioleiomyomatosis and is suggested for use in other soft tissue sarcomas [2,3,4]. Temsirolimus has largely been approved for the same tumors and was the first rapamycin analog approved for use in advanced Renal Cell Carcinoma [5]. Everolimus has been approved for use in advanced hormone-receptor-positive, HER2-negative breast cancer, renal cell cancer, neuroendocrine tumors, and subependymal giant cell astrocytoma among other cancers [6,7,8]. Ridaforolimus, although still in clinical trials, has been tested for the treatment of many of the same cancers along with prostate, endometrial, lung, and hematological malignancies [9]. Many of these agents have also been considered for a wide range of lymphomas [10]. The common thread that seems to be emerging for treatment with these agents is that they are useful in tumors with mutations in the tuberous sclerosis complex and perhaps other alterations of the mammalian target of rapamycin pathway [11,12,13]. Another common theme is that they may be more effective in combination therapy [12,14,15].

Much of the research into the personalization of rapamycin and its analogs have centered around understanding the characteristics of tumors that respond well to treatment. This review summarizes the molecular properties of susceptible tumors. This review also investigates how the body metabolizes these agents and whether human variation may play a role in the efficacy of the rapalogues in individuals. Published data further points out how individual tumors may possess resistance to these drugs before treatment even begins. The net effect is that there are at least three layers of molecular analysis required to fully match the drugs to tumors that will likely be susceptible to their anti-tumor activity.

## 2. Materials and Methods

The current narrative review aims to examine the evidence on mTOR inhibitors metabolism and uses as an immunosuppressant and chemotherapeutic drug to provide an approach for personalized medicine. A literature search was conducted using PubMed and the Google Scholar database for published articles that have examined the FDA-approved uses of mTOR inhibitors, how they are metabolized in the body, and mTOR function. Terms used were “mTOR inhibitors”, “Everolimus”, “Sirolimus”, “Temsirolimus” or “rapalogues” in combination with “mechanism of action”, “metabolism”, “pharmacokinetics”, and “efficacy”. Further articles were found through cross-referencing. Primary studies (e.g., retrospective studies, prospective studies, observational studies, randomized controlled trials, etc.), basic science research, metanalyses, and systematic reviews were included.

The gene expression data came from multiple published datasets. We identified datasets with tumor or normal samples and raw CEL files from microarrays run on U133 Plus 2.0 arrays available for download. This CEL file data was downloaded from the microarray databases Gene Expression Omnibus at the National Center for Biotechnology Information: GSE7307—Human Body Index—arrays for normal tissue samples.

- GSE2109—Expression project for Oncology—many tumor samples from various pathological groups;
- GSE11151—Kidney;
- GSE12102—Sarcoma;
- GSE13433—Sarcoma;
- GSE14827—Sarcoma;

and the European Bioinformatics Institute:

- E-MEXP-964—Sarcoma.

The data were placed into the Affymetrix Expression Console software and processed with the MAS 5.0 algorithm to calculate signal intensities for each probeset on each array using a trimmed mean average of 500 to scale all samples. The sample data quality was then assessed using the Expression Console reports, R QC reports, and an RNA quality analysis tool developed at the Moffitt Cancer Center. Samples were rejected for having high scaling factors (>12), low percent present calls (<35), high RNA quality scores (>4.0), or odd-looking scatter plots when compared to a reference array.

The data was further screened for duplicate arrays and misleading or confusing clinical annotations. For example, some samples were removed because they were clinically annotated from females but contained gene expression from the Y chromosome. Other problems included samples labeled both as tumors and as normal tissue or with annotations that were not clearly metastatic when the site of origin did not match the pathological diagnosis. Following data preprocessing the sample data was loaded into Excel files based on tumor type. The probeset information from the array data was reduced to identify one representative probeset for each gene. Where multiple probesets detected the same gene the probeset that yielded the largest average expression value was selected to represent that gene and all others were discarded.

The normal tissue samples were processed in the same manner. Four representative samples from each tissue site were selected to be included in the final collection of normal tissues. The normal tissue sites were selected from a larger set of normal tissues around the body to contain tissues where some cytochrome gene expression has been known to occur. The normal samples and tumor samples were processed in parallel to ensure that relative expression would be accurate.

## 3. Results

### 3.1. The First Level of Personalized Medicine: Tumor Mutations Related to Mechanism of Action

**Figure 1:**
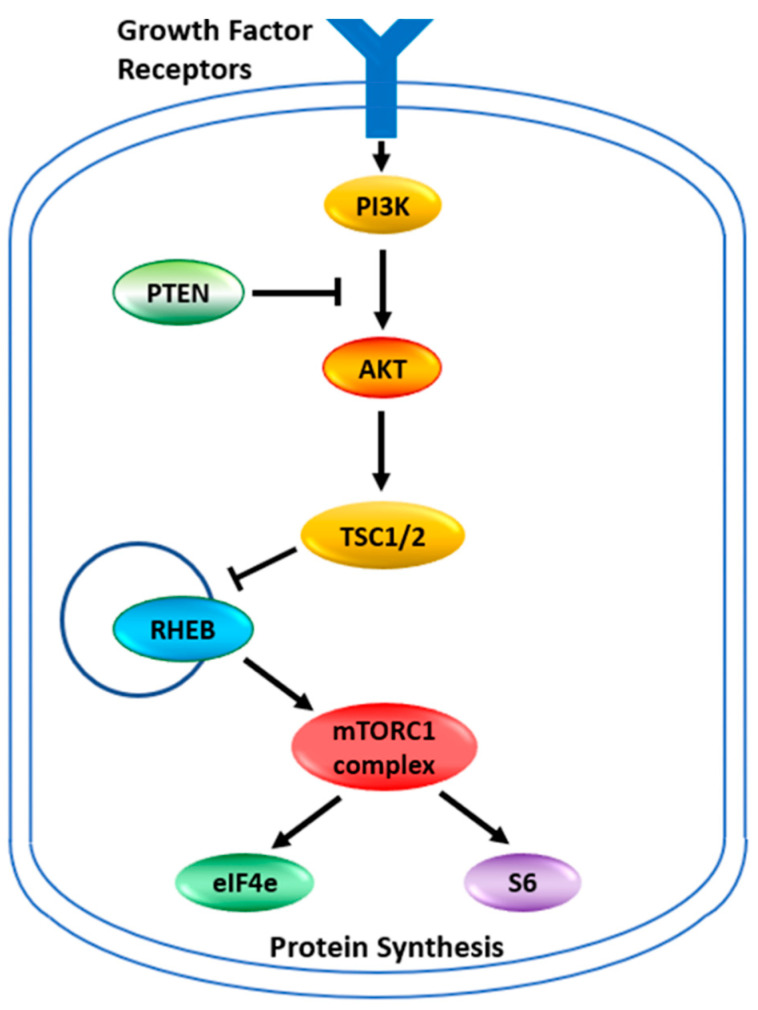
The mTOR pathway. Growth factor signaling leads to the activation of cell growth. Signaling pathways are networks rather than linear pathways, but the key players identified in the mTOR pathway are indicated. PI3K—Phosphoinositide 3-kinase; PTEN—Phosphatase and tensin homolog; AKT—AKT serine/threonine kinase 1; TSC—tuberous sclerosis complex is also known as the hamartin-tuberin complex; RHEB—Ras homolog, mTORC1 binding; eIF4e—eukaryotic translation initiation factor 4E; S6—ribosomal protein S6 kinase B1. The TSC complex is a tumor suppressor that blocks the release of RHEB. Free RHEB binds GTP and activates the mTORC1 complex which in turn phosphorylates S6 and the repressor protein 4E-BP1 releasing eiF4e. S6 and eiF4e go on to stimulate protein synthesis at different places in the process.

The target for the rapalogues is the mammalian Target of Rapamycin, now known as the mechanistic Target of Rapamycin kinase (mTOR). It is a serine/threonine protein kinase in the PI3K kinase family. It forms two main complexes in the cell known as mTOR complex 1 (mTORC1) and mTOR complex 2 (mTORC2) [16]. These complexes have a wide variety of functions within a cell, mainly involved in regulating growth through nutrient metabolism and protein synthesis [15]. Complex 1, in addition to stimulating protein synthesis through phosphorylations of the complexes containing the eukaryotic translation initiation factor 4E binding protein 1 and ribosomal protein S6 kinase, also stimulates the synthesis of new ribosomes [15]. Through this complex, mTOR also plays a role in glucose metabolism, upregulating genes that allow cancer cells to utilize glucose under anaerobic conditions [15]. The abbreviated pathway for activation of mTORC1 or mTORC2 is illustrated in Figure 1. Under normal circumstances, growth factors activate PI3K which produces phosphatidyl-inositol,3,4,5 triphosphate (PIP3). This activates a number of proteins including AKT, which phosphorylates TSC2, inhibiting the tuberous sclerosis complex. PTEN dephosphorylates PIP3 thus inhibiting the activation pathway. With the tuberous sclerosis complex inactivated the RHEB GTPase now becomes active to induce mTORC1. The mTORC2 complex is more directly activated by PIP3. Between the two complexes, many cellular pathways are stimulated to support cellular growth. This pathway of AKT/PTEN inactivating the tuberous sclerosis complex, thus leading to the activation of mTOR and thereby increasing glucose metabolism and protein synthesis is believed to be critical for allowing tumor growth under lower nutrient and anaerobic conditions. The activation of mTOR in cancers can occur through the loss of PTEN function or increased AKT expression [15,17,18]. The reduced activity of the Tuberous Sclerosis Complex, which is a tumor suppressor, is increasingly recognized as a key mechanism for cancer. Mutations in the TSC1 and TSC2 genes of these complexes also activate the mTOR pathway and induce cancers [13]. This makes this cellular growth pathway an attractive therapeutic target. It is also why tumors with mutations in AKT, PTEN, TSC1, and TSC2 are now considered indicators for treatment with the rapamycin analogs that inhibit this pathway [8,11,13,15,18,19]. It has been shown that tumors with mutations within this pathway are sensitive to rapamycin and its analogs [20]. Any mutation in this pathway should be the first indication that the rapalogues might be a useful therapeutic choice.

#### Inhibition of the mTOR Pathway

**Figure 2:**
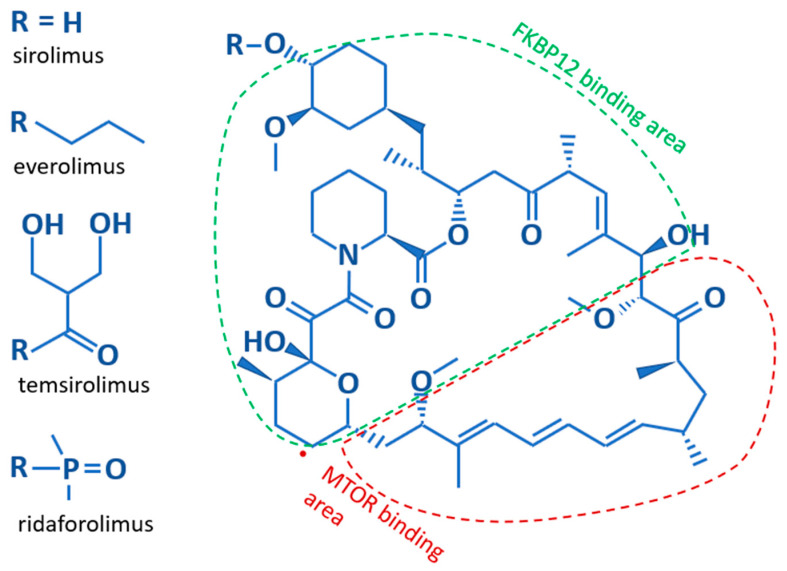
The basic structure of Sirolimus and its analogs. The core structure is identical for all rapalogues. The difference lies in their side chains; listed along the left side. Additionally indicated is which parts of the core structure bind to the FKBP12 and mTOR proteins.

Structurally, Sirolimus and its analogs contain a core lipophilic macrocyclic lactone, which is more soluble in organic solvents than water (Figure 2). The difference between the various analogs results from substitutions for the hydroxyl group on the C40 carbon of the core structure. These substitutions affect the bioavailability, metabolism, and half-life of the analogs [13]. Sirolimus, with a hydroxyl group on C40, and Everolimus, which bears a stable 2-hydroxyethyl chain, both have low solubility making them orally administered medications. The hydrophobicity of Sirolimus has also made it available for use as a topical agent [21,22]. Temsirolimus contains a 2,2-bis-(hydroxymethyl)-propionate substitution providing higher water solubility, as does the phosphate group on Ridaforolimus allowing these rapalogues to be administered by infusion. Further differences are illustrated in Table 1. The core macrocyclic lactone binds mTOR allosterically in complex with the FK506-binding protein 12 (FKBP12). Where the two proteins interact with the core structure of Sirolimus is shown in Figure 2. It appears that all rapalogues interact with the mTORC1 complex in the same way [14]. This binding inhibits the activity of the mTOR complex1, effectively removing it from the functional pool. Blocking mTOR creates a starvation-like effect in cancer cells, interfering with cell growth, division, metabolism, and angiogenesis. The mTORC2 complex is not directly affected but may be reduced when chronic exposure to a rapalogue leads to reduced availability of mTOR complex components [23]. The partial inhibition of the mTORC2 complex may be the reason that chronic exposure is better than transient exposure during treatment and why rapalogues may not be completely effective as single agents during treatment.

### 3.2. The Second Level of Personalized Medicine: Metabolism of Rapalogues

Sirolimus and its derivative compounds are some of the most easily tolerated therapeutic drugs for cancer treatment. Sirolimus is routinely used long-term for the suppression of the immune system in transplant patients [24]. However, the doses used to prevent graft rejection are lower than those used for cancer treatment. At higher doses, these compounds do show toxicity at certain doses and in certain patients. For this reason, pharmacokinetic monitoring of the drug is often recommended during treatment [25,26,27]. All of the rapalogues reach peak concentrations quickly, infiltrate tissues easily, and have relatively long half-lives in vivo [26,28]. This does not mean that they function equally in all persons. In addition to influencing the route of administration, the side chains influence the metabolism of these drugs in vivo. However, not all of the mechanisms by which the rapalogues are metabolized have been worked out and the underlying reason for differences remains unknown. For example, Sirolimus and Everolimus appear to be transported in and out of cells by slightly different mechanisms [29]. In addition, Temsirolimus is often referred to as a pro-drug, which must be converted to Sirolimus to function [30,31,32] whereas others claim that Temsirolimus can bind FKBP12 and the mTOR complex [14,33]. It is known that Temsirolimus can be converted to Sirolimus by the action of CYP3A4, an enzyme that can further metabolize the rapamycin derivatives to many other compounds. The current data suggests that all rapalogues are mainly metabolized by the same enzymes (Table 1). The bulk of the metabolism occurs in the liver by cytochrome family members. The major players seem to be CYP3A4, CYP3A5, and CYP2C8 with the major transporter ABCB1, also known as p-glycoprotein or the multi-drug resistance protein MDR1 [26,28,30,32,34].

The members of the cytochrome family have long been known as the key players in the metabolism of many drugs and CYP3A4 and 3A5 are among the enzymes most commonly involved in drug metabolism [35,36]. These enzymes are similar and are often described as one entity 3A4/5, but there is also controversy about how much each contributes to the metabolism of various drugs [37]. The role these enzymes play in the efficacy and toxicity of the rapalogues has some individuals calling for monitoring blood concentrations during treatment as well as reviewing co-administered drugs that might interfere with the metabolism of the rapalogues [33,38,39]. However, personalization of treatment should also consider genetic testing of the patient. CPIC and PharmGKB recognize that 17 human variants influence the activity of CYP3A4, 9 variants that influence the activity of CYP3A5, and 16 variants of CYP2C8 [40,41,42,43]. More importantly, up to 30% of people carry at least one of these variants. These variants influence the rate of drug metabolism and therefore can influence the active dose achieved within the tumor or cause toxicity in some patients. Pharmacogenetics is gaining credibility as important for the management of rapalogues within transplant patients [44,45,46,47]. It is just beginning to be recognized within the oncology setting [39,48,49]. Proper care should begin with a pharmacogenomic analysis of the patient. Although the full spectrum of human variants that influence the metabolism of Sirolimus is not yet known, enough variants are currently known with a high enough frequency in the human population to establish dosing adjustments or at least detect genotypes at risk for toxicities that might disrupt treatment. Pharmacogenetic analysis should be supplemented with pharmacokinetic monitoring to detect metabolic problems not predicted by the genetic analysis. Finally, the oncologist should also review the full course of medications taken by the patient while on one of the rapalogues to assess whether drug interactions could influence the metabolism further.

### 3.3. The Third Level of Personalized Medicine: Metabolism of Rapalogues by Tumor Cells

Effective chemotherapeutic treatment depends on reaching an effective concentration of the active compound within the tumor cells. This depends on multiple factors including, drug absorption, systemic distribution, protein binding, drug metabolism (activating or inactivating), transport in and out of tumor cells, and ultimately, excretion from the body. There is now one more factor to consider. Drug metabolism within the tumor.

**Figure 3:**
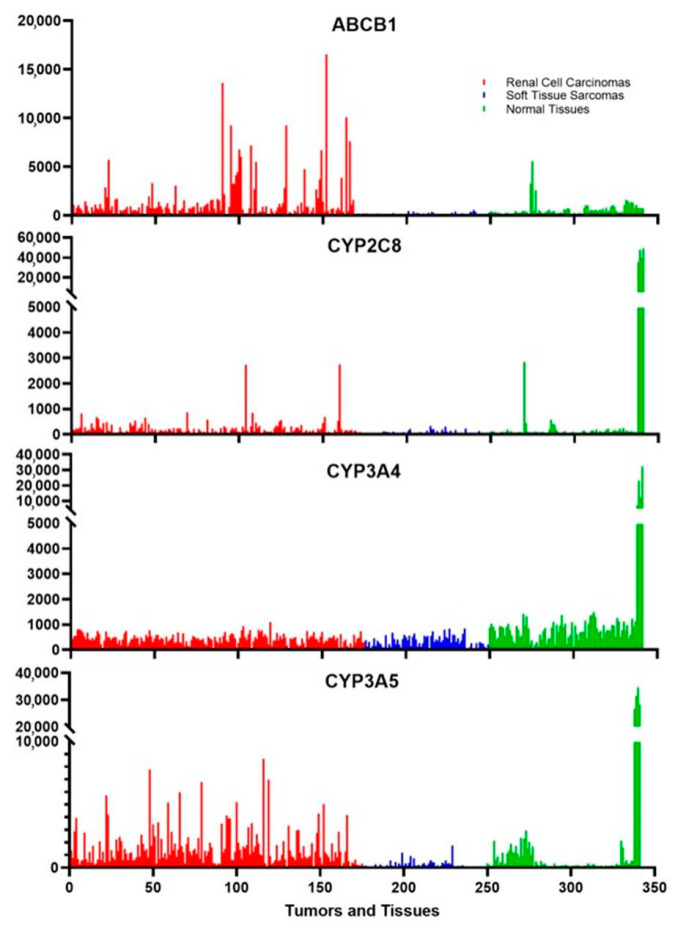
The expression of metabolizing enzymes and transporters of the rapalogues in tumors is commonly treated by these chemotherapeutic agents. ABCB1 is the multidrug resistance protein, and the cytochromes are the main three enzymes that metabolize the rapalogues. The red samples are 175 different Renal cell carcinomas, the blue samples are 74 different soft tissue sarcomas, and the green samples represent 4 samples each of 24 different normal tissues of the body. The last four samples are liver samples, which generally have the highest expression of any tissue. The eight samples just prior are from the medulla and cortex of the kidney, which can be used for comparison to the renal cell carcinoma samples.

Drug metabolism occurs mainly in the liver, but other tissues also express low levels of certain cytochromes. These tissues include parts of the intestines and the kidneys; tissues that may be exposed to xenobiotics. Figure 3 shows that there is a very high expression of CYP3A4, CYP3A5, and CYP2C8 in liver tissues, as expected, and CYP3A4 is measurably expressed in many other normal tissues. In contrast, CYP3A5 is expressed in kidney cells and a few other tissues while CYP2C8 is weakly expressed, if at all, in tissues other than the liver except for an occasional peak expression for unknown reasons. Two of the cancer classes that the rapalogues are approved to treat are renal cell carcinomas and soft tissue sarcomas. Figure 3 shows that there is a low level of expression of CYP3A4 in both of these cancers. More surprising is the fact that some renal cell carcinomas express CYP3A5 at a level much higher than those found in normal tissues other than the liver. CYP2C8 is also expressed in certain renal cell carcinomas. These three enzymes modify the rapalogues and reduce the amount of active compound. The levels of expression indicate that certain tumors might be partially resistant to the rapalogues due to the metabolism of the drugs within the tumors. This could explain why some tumors shrink and others grow under the same treatment conditions.

Something that is well documented to influence chemotherapeutic success is the multidrug resistance protein [50]. Also known as p-glycoprotein, ABCB1 is not highly expressed in normal tissues (Figure 3). In contrast, this gene is very highly expressed in some renal cell carcinomas (Figure 3). This gene is not expressed in some tumors, lightly expressed in others, and very highly expressed in some tumors. This likely leads to a highly varied response to the rapalogues. This multidrug transporter is an ATP-dependent efflux pump with broad substrate specificity, which can influence the activity of many chemotherapeutic agents. The transport of the rapalogues out of the tumors is influenced by both the amount of ABCB1 in the tumor cells and possibly by other drugs taken by the patient that are also transported out of cells by ABCB1.

The point of talking about these enzymes and transporters is that any given tumor of a pathological class could be well-equipped to counteract the drug chosen to treat that tumor. This calls for a gene expression analysis of the tumor prior to the final selection of the treatment choice. This is a suggested third level of personalization that could increase the efficacy of treatment. Unfortunately, all of the members of the rapamycin homologs are influenced by the same metabolizing enzymes. If a tumor is resistant to one, it is likely resistant to other members of this class. The wrong combination of enzymes in the tumor might signal a need to shift to a different class of drugs to treat the tumor. The question shifts to whether there are alternatives that target a different point in the mTOR pathway.

#### Alternatives to Rapalogues

Currently, there are a number of drugs in development that target various aspects of the AKT/PTEN pathway through mTOR. Table 2 shows some of them. None of these have yet reached clinical approval. Some of those listed have been discontinued or suspended in development while others are very early in the long process toward clinical approval [51]. Some are quite broad in their activity, hitting several cellular targets while others show more specificity for targets in the AKT/PTEN pathway. There is clearly more work to conduct to determine the safety of these agents, find the correct dosing, and establish that tumors dependent on the AKT/PTEN pathway respond to their use. So, it may be some time before there is an FDA-approved treatment plan for some of these alternatives that might complement the rapalogues. The optimism here is that alternatives exist and thus there is hope for those patients for whom a molecular analysis rules out the rapalogues but indicates that the mTOR pathway should be targeted.

## 4. Discussion

With the transition to precision oncology, there is growing support for a shift from treating tumors based on their anatomical site to treatment based on the molecular defects driving their growth [52,53,54]. With agnostic drug development and umbrella trials, it will become more common to see drugs, such as the rapalogues, used in a wider variety of tumors with respect to their anatomical site and a narrower spectrum of tumors, based on their molecular profile. Targeting the mTOR complexes and the pathways that activate them has a track record of success. What must next be incorporated into the decision tree is the genetic profile of the patient so that one can account for a patient’s ability to tolerate the treatment. CYP3A4, CYP3A5, and CYP2C8 have known variants that affect the activity and could explain why these agents are toxic for some patients but not in others [39,40]. There are known alleles of CYP3A4 that should compromise 3A4 activity against the rapalogues in approximately 15% of people depending on their heritage and for 3A5 roughly 12% of the population. This accounts for just the most common variants with poor activity. There are many others [55]. The clinically validated effects of CYP2C8 and ABCB1 variants are yet to be determined. There is far more research required before we know the whole story about how human variation influences efficacy and toxicity.

For most drugs, the phase I cytochrome family of enzymes only explains a proportion of the drug’s metabolism and known human variants explain only about a third of the variability shown in people. Further work is required to understand the breakdown and excretion of the rapalogues so that the full extent of the role that each enzyme, transporter, and binding protein plays in the amount of drug presented to a tumor and how drug levels relate to toxicity. Any new genes shown to be involved in metabolism will also require an assessment of human variation for that gene. Additional work is also required on the genes mentioned in this review. This includes finding new variants that impact activity, identifying whether the altered activity impacts all drugs to a similar extent, and establishing what the impact means in terms of using the rapalogues. Some variants may be tolerable in the heterozygous state but detrimental as homozygotes. Some variants may require an adjustment in dosage while others may require switching to an alternative drug. How the allele composition of a patient relates to the ultimate medical solution will require some carefully planned clinical trials. A large amount of information is known about many of the cytochromes as they play a role in many pharmaceuticals, but little is known about other enzymes such as ABCB1. The ultimate goal is to be able to translate a genotype into a phenotype and then into a medical recommendation for a patient about to be treated with Sirolimus or one of its derivatives. For now, it is important to incorporate what is currently known about CYP3A4, CYP3A5, CYP2C8, and ABCB1 into the assessment of a patient when the mTOR pathway is a desired target in treating a tumor.

There is one more level of assessment that can be currently implemented to improve the efficacy of treatment with the rapalogues. A gene expression analysis of the tumor should be performed. A whole genome analysis should be performed one day as many more things will likely be important to know about tumor gene expression, but for now, a tumor should be analyzed for the expression of proteins that could impact the response or resistance of tumors to the rapalogues. It is well known that gene expression analysis can be performed from a small amount of excised tumor or a core needle biopsy [56]. This should be done early and could be performed at the same time that the tumor genome is analyzed to know that one would be considering the use of a rapalogue. With respect to the rapalogues, many of the currently treated tumors express enzymes that can break down the drugs or export them from tumor cells. Such tumors are less likely to respond favorably to treatment. In these cases, one would possibly select a different drug that might target another part of the mTOR pathway or co-administer a second drug that interferes with the tumor metabolism of the rapalogues. To know for certain which option has the best outcome will require more clinical trials. Thus, there is a large amount of research still required to optimize the use of Sirolimus, Everolimus, and Temsirolimus.

## 5. Conclusions

Oncology is leading the medical community in personalizing treatment options. Whether it be the development of specifically targeted agents or the use of genotype-guided dosing, the cancer community has accepted the concept that individual tumors might require individualized treatment. With the rapalogues, a picture is emerging that tumors dependent on the mTORC1 pathway respond well to these agents. But the response to these agents is still variable. There are two different forms of molecular analysis that could reveal features of the patient and the tumor that explain this variability. There is sufficient information about the breakdown of rapalogues to suggest that patients be genotyped to help predict tolerance by individual patients and that tumors be analyzed for the expression of resistance mechanisms that might reduce the efficacy of the treatment. In the near future, these additional molecular analyses will likely be required to fully personalize this treatment option.

## Figures and Tables

**Table 1:** Rapalogue family of agents, dosing, and the enzymes that metabolize them.

Rapalogue	Sirolimus	Everolimus	Temsirolimus	Ridaforolimus
Brand Name	Rapamune	Afinitor and others	Torisel and others	Deforolimus
Active Compound	Sirolimus	Everolimus	Sirolimus *	Ridaforolimus
Route of administration	Oral	Oral	Infusion	Oral or Infusion
Frequency	Once Daily	Once Daily	Once Weekly	Either
Metabolizing enzymes	CYP3A4, CYP3A5, CYP2C8, P-glycoprotein	CYP3A4, CYP3A5, CYP2C8	CYP3A4, CYP3A5, CYP2C8, CYP2B6	CYP3A4, P-glycoprotein

* Some report that Temsirolimus also can bind to mTOR and inhibit activity. Table adapted from MacKeigan and Krueger [13].

**Table 2:** New therapeutic agents that target other points in the mTOR pathway.

ATP-Competitive Inhibitors/Pan TOR Inhibitors	Dual mTOR/PI3K Inhibitors
AZD8055	Dactolisib
vistusertib	BGT226
sapanisertib	Voxtalisib
OSI027	Apitolisib
Onatasertib	gedatolisib
WYE-125132	sf1126
Torkinib	PF04691502
PKI402	Omipalisib
Palomid 529	CMG002
	GNE-477
	Bimiralisib

## Data Availability

The data used in this manuscript is from previously published work. The gene expression data can be obtained from the sources indicated in the Materials and Methods section.
